# Confidence interval construction for multivariable truncated spline logistic model (MTSLM)

**DOI:** 10.1016/j.mex.2026.103959

**Published:** 2026-05-15

**Authors:** Afiqah Saffa Suriaslan, I Nyoman Budiantara, Vita Ratnasari, Dursun Aydin

**Affiliations:** a Departement of Statistics, Institut Teknologi Sepuluh Nopember, Kampus ITS-Sukolilo, Surabaya 60111, Indonesia; b Department of Statistics, Faculty of Science, Mugla Sitki Kocman University, Mugla 4800, Turkey

**Keywords:** Truncated spline, Logistic regression, Convidence interval, Pivotal quantity, Human development Index

## Abstract

This study highlights the construction of confidence intervals within the Multivariable Truncated Spline Logistic Model (MTSLM). Its truncated spline function allow flexible modeling of nonlinear relationships across sub-intervals that conventional logistic regression cannot capture. A simulation study is conducted to validate the performance of proposed method, and Human Development Index (HDI) data in Indonesia are used as an empirical illustration to demonstrate its practical applicability. The results show that several predictors are statistically significant. For instance, access to clean water has a confidence interval [0.0124, 0.0566], while the percentage of poor population shows interval with range [−0.4039, −0.0341]. Nonlinear effects are evident in the unemployment variable, with both confidence interval [0.5083, 1.2985] and [−1.4760, −0.3656]. Similar patterns are observed for school enrollment rates. The confidence interval analysis result provides insight into the reliability of these predictor effects, emphasizing the role of infrastructure, education, and social-economic factors.The findings demonstrate that MTSLM not only improves accuracy but also provides informative and reliable estimates of predictors influencing HDI.

The highlights of this study:

• Develop convidence interval for MTSLM to flexibly nonlinear model.
• A simulation study and real HDI data are used to validate the proposed method.
• The MTSLM model outperforms binary logistic model.

## Graphical abstract

**Figure:**
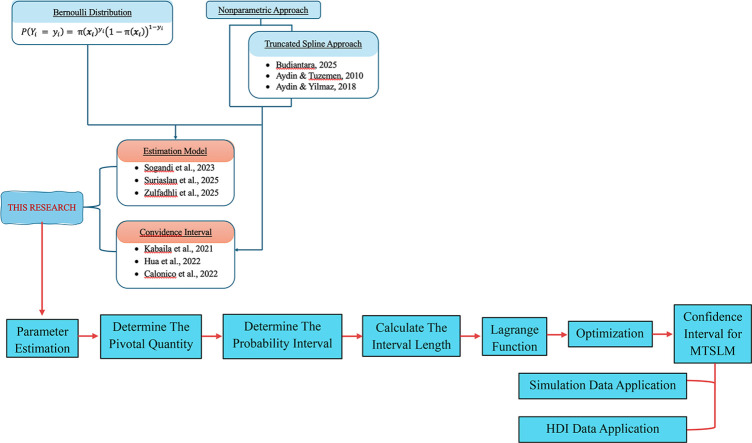

## Specifications table

**Table:** 

Subject area	Mathematics and Statistics
More specific subject area	Statistics; Statistical Inference; Nonparametric Regression; Binary Outcomes Data
Name of your method	Multivariable Truncated Spline Logistic Model (MTSLM)
Name and reference of original method	Spline Function in the Proceedings of the Edinburgh Mathematical Society of Whittaker, E. T. (1922), On a New Method of Graduation, 41, 63–75.
	doi: https://doi.org/10.1017/S0013091500077853
	Maximum likelihood estimation in the book of Hosmer, D. W., & Lemeshow, S. (2000), Applied Logistic Regression, 2nd Edition. United States of America, Canada. https://books.google.co.id/books?id=Po0RLQ7USIMC&lpg=PP1&pg=PP1%23v=onepage&q&f=false#v=onepage&q&f=false
Resource availability	This study uses secondary data from publications issued by the Indonesian Central Statistics Agency (BPS) (https://www.bps.go.id/id/statistics-table/2/NDEzIzI=/-metode-baru–indeks-pembangunan-manusia.html)
	To facilitate the implementation of the methods described in this study, we developed a package (syntax) using R Studio. We have made this package publicly available for ease of use with various datasets. This package can be accessed via the following link: https://github.com/Afiqah-ctrl/Convidence-Interval-for-MTSLM.git

## Background

In statistical analysis, the Bernoulli distribution is one of the most fundamental discrete probability distributions for modelling experiments with two possible outcomes. Suppose $Y_{i}$ is a random variable that takes the value 1 for a “success” and 0 for a “failure.” The corresponding probability function is given by [1]:

(1) $$P\left(Y_{i}\;=\;y_{i}\right)=\;\pi {\left(x_{i}\right)}^{y_{i}}{\left(1-\pi \left(x_{i}\right)\right)}^{1-y_{i}},\;y_{i}=\left\{0,1\right\},\;0<\pi \left(x_{i}\right)<1,i=1,2,\ldots ,n$$

where $\pi \left(x_{i}\right)=P\left(Y_{i}=1|x_{i}\right)$ denotes the success probability for the i=th observation.

Although the Bernoulli distribution is discrete and generally asymmetric, a normal distribution approximation can be used under certain conditions. According to the Central Limit Theorem (CLT), the sum or mean of a large number of independent Bernoulli random variables converges to a normal distribution [2]. This approximation is widely used in statistical inference for binary data when the sample size is large enough and the probability values are not close to the boundaries of 0 or 1.

The Bernoulli distribution has been extensively applied across a wide range of disciplines. In the health sector, it has been used in risk adjusted control charts for multistage healthcare monitoring [3], to integrate high quality scientific evidence with virtual patient case studies [4] and studies on diabetes [5]. In the Indonesia context, this distribution has been applied in various economic studies, such us poverty modelling [6], unmet needs analysis [7,8], and to study aspects of the Human Development Index (HDI) [9].

HDI is a composite indicator measuring human life through health, education, and decent living standards. HDI values can be categorized into high and low groups, enabling the application of binary outcome models [10]. However, the relationship between HDI and its predictor variables often exhibits nonlinear patterns. Consequently, flexible modelling approaches are required [11]. The truncated spline method provides a suitable alternative because it allows changes in relationship patterns at specific points, known as knots, without requiring a predetermined functional form [10,12,13]. Thus, the use of HDI cases in this study serves as a means to demonstrate the effectiveness of truncated spline based modelling in handling binary data with nonlinear relationship structures.

Statistical inference such as confidence intervals, is employed to assess predictor effects and quantify uncertainty in parameter estimates within binary outcome models. The construction and theoretical properties of confidence intervals have been widely studied, including confidence intervals using Durbin–Watson statistics [14], resampling based confidence intervals for step regression [15], and advanced inferential properties in nonparametric regression frameworks [16].

This study aims to develop a confidence interval construction method for Multivariable Truncated Spline Logistic Model (MTSLM). The proposed approach integrates truncated spline representations into the logistic likelihood function and derives confidence intervals based on the asymptotic properties of the maximum likelihood estimator. The main advantage of this method is its ability to quantify estimation uncertainty while preserving nonparametric modelling flexibility. Compared to conventional logistic regression, it more effectively captures nonlinear predictor effects and provides explicit interval inference. Methodologically, this work extends statistical inference beyond point estimation, strengthening the theoretical foundation of truncated spline logistic model. The method is illustrated using HDI data categorized into high and low levels.

## Method details

### Multivariable truncated spline logistic model (MTSLM)

The key idea of this section is to utilize the logit link function to transform the nonlinear relationship between predictor variables and success probabilities into a linear structure on the logit scale. This transformation is essential because the success probability $\pi (x_{i})$ is bounded between 0 and 1, whereas the logit function is defined over the entire real line. The relationship is expressed as follows [6]:

(2) $$logit\left(\pi \left(x_{i}\right)\right)=log\left(\frac{\pi \left(x_{i}\right)}{1-\pi \left(x_{i}\right)}\right)=f\left(x_{1i},\;x_{2i},\;\ldots ,\;x_{pi}\right)$$

Here, the unknown function $f(x_{1i},\;x_{2i},\;\ldots ,\;x_{pi})$ is approximated using a multivariable truncated spline function of degree m. The explicit logit equation for the model is defined as:

(3) $$logit\left(\pi \left(x_{i}\right)\right)=\beta_{0}+\;\sum_{j=1}^{p}\sum_{k=1}^{m}\beta_{jk}x_{ji}^{k}+\;\sum_{j=1}^{p}\sum_{u=1}^{r}\beta_{j\left(m+u\right)}{\left(x_{ji}-\;K_{ju}\right)}_{+}^{m}\;$$

where $\beta_{0},\;\beta_{jk},$ and $\beta_{j(m+u)}$ are the unknown coefficients to be estimated and m≥1 denotes the degree of the spline, $K_{ju}$ represents the u-th knot associated with the j-th predictor, and ${\left(.\right)}_{+}^{m}$ represents the truncated spline basis function of degree m. Note that the truncated power basis function in (3) is defined aswhere u=1,2,…,r indicates the number of knot points.

For computational implementation, the model (3) is represented in matrix notation as:

(4) $$logit\left(\pi \right)=X\beta =1_{n}\beta_{0}+\sum_{j=1}^{p}x_{j}\beta_{j}=\left(\begin{array}{ccc} 1_{n} & x_{1} & \begin{array}{cc} \cdots & x_{p} \end{array} \end{array}\right)\left(\begin{array}{c} \beta_{0}\\ \beta_{1}\\ \begin{array}{c} \vdots \\ \beta_{p} \end{array} \end{array}\right)$$

where $1_{n}$ denotes an n×1 vector of ones, while $x_{j}$ presents the truncated spline basis matrix for the j-th explanatory variable, given by

$$x_{j}={\left[\begin{array}{ccc} 1 & x_{i1} & \begin{array}{ccc} \ldots & x_{i1}^{m} & \begin{array}{ccc} {\left(x_{i1}-\;K_{j1}\right)}_{+}^{m} & \ldots & {\left(x_{i1}-\;K_{jr}\right)}_{+}^{m} \end{array} \end{array} \end{array}\right]}_{i=1}^{n}$$

The corresponding parameter vector can be expressed as

$$\beta_{j}={\left[\begin{array}{ccc} \beta_{0} & \;\beta_{j1} & \begin{array}{ccc} \ldots & \beta_{jm} & \begin{array}{ccc} \;\beta_{j\left(m+1\right)} & \ldots & \beta_{j\left(m+r\right)} \end{array} \end{array} \end{array}\right]}^{\prime }.$$

Hence, the i-row of the design full matrix $X\in R^{n\times \;[1\;+\;p(m+r)]\;}$is

$$X_{i}=\left[1,x_{1i},\ldots ,x_{1i}^{m},{\left(x_{1i}-\;K_{11}\right)}_{+}^{m},\ldots ,{\left(x_{1i}-\;K_{1r}\right)}_{+}^{m},\ldots ,x_{pi},\ldots ,x_{pi}^{m},\;{\left(x_{pi}-\;K_{p1}\right)}_{+}^{m},\ldots ,{\left(x_{pi}-\;K_{pr}\right)}_{+}^{m}\right]$$

Accordingly, the associated parameter vector $\beta \in R^{[p(m+r)+1\;]\times \;1\;}$ is

$$\beta ={\left[\beta_{0},\;\beta_{11},\ldots ,\beta_{1m},\;\beta_{1\left(m+1\right)},\ldots ,\beta_{1\left(m+r\right)},\ldots ,\beta_{p1},\ldots ,\beta_{pm},\;\;\beta_{p\left(m+1\right)},\ldots ,\beta_{p\left(m+r\right)}\right]}^{\prime }.$$

Following the construction of the spline based design matrix, the MTSLM is finalized for binary outcome data. By applying the inverse logit transformation (3), the success probability $\pi (x_{i})$ for the i-th observation is estimated as

(5) $$\pi \left(x_{i}\right)=\frac{\exp(\beta_{0}+\;\sum_{j=1}^{p}\sum_{k=1}^{m}\beta_{jk}x_{ji}^{k}+\;\sum_{j=1}^{p}\sum_{u=1}^{r}\beta_{j\left(m+u\right)}{\left(x_{ji}-\;K_{ju}\right)}_{+}^{m}\;)}{1+\;\exp(\beta_{0}+\;\sum_{j=1}^{p}\sum_{k=1}^{m}\beta_{jk}x_{ji}^{k}+\;\sum_{j=1}^{p}\sum_{u=1}^{r}\beta_{j\left(m+u\right)}{\left(x_{ji}-\;K_{ju}\right)}_{+}^{m}\;)}=\frac{\exp\left(X_{i}^{\prime }\beta \right)}{1+\exp\left(X_{i}^{\prime }\beta \right)}$$

Assuming that the outcome variable $Y_{i}$ follows a Bernoulli distribution with success probability $\pi (x_{i})$, the unknown parameters are estimated using the Maximum Likelihood Estimation (MLE) method. The likelihood function is given by

(6) $$l\left(\beta \right)=\;\prod_{i=1}^{n}P\left(Y_{i}=\;y_{i}\right)=\prod_{i=1}^{n}\pi {\left(x_{i}\right)}^{y_{i}}{\left(1-\pi \left(x_{i}\right)\right)}^{1-y_{i}}$$

where $\pi_{i}\left(x_{i}\right)=P\left(Y_{i}=1|x_{i}\right)$, as defined in (1).

For computational convenience, the logarithm of the likelihood function is maximized. Taking logarithms of (6), the log likelihood function can be written as

(7) $$\begin{array}{c} L\left(\beta \right)=\sum_{i=1}^{n}\left[y_{i}\log\pi \left(x_{i}\right)+\left(1-y_{i}\right)\log\left(1-\pi \left(x_{i}\right)\right)\right]\\ =\sum_{i=1}^{n}\left[y_{i}\left(f\left(x_{1i},\;\ldots ,\;x_{pi}\right)\right)-\log\left\{1+\exp(f\left(x_{1i},\;\ldots ,\;x_{pi}\right))\right\}\right] \end{array}$$

where $f\left(x_{i}\right)=f\left(x_{1i},x_{2i},...x_{pi}\right)=X_{i}^{\prime }\beta ,\;and\;\pi \left(x_{i}\right)=\exp\left\{f(x_{i})\right\}/\left(1+\exp\{f(x_{i})\}\right)$.

The MLE of the parameter vector is obtained numerically using the Newton–Raphson iterative scheme, expressed as

(8) $${\hat{\beta }}^{\left(t+1\right)}=\;{\hat{\beta }}^{\left(t\right)}-\;{\left(H{\left(\hat{\beta }\right)}^{\left(t\right)}\right)}^{-1}g{\left(\hat{\beta }\right)}^{\left(t\right)}$$

Here,

$$\hat{\beta }={\left[{\hat{\beta }}_{0},\;{\hat{\beta }}_{11},\ldots ,{\hat{\beta }}_{1m},\;{\hat{\beta }}_{1\left(m+1\right)},\ldots ,{\hat{\beta }}_{1\left(m+r\right)},\ldots ,{\hat{\beta }}_{p1},\ldots ,{\hat{\beta }}_{pm},\;\;{\hat{\beta }}_{p\left(m+1\right)},\ldots ,{\hat{\beta }}_{p\left(m+r\right)}\right]}^{\prime }$$

$$g{\left(\hat{\beta }\right)}^{\left(t\right)}=\;\frac{\partial L\left(\beta \right)}{\partial \beta },\text{is}\;\text{the}\;\text{gradient}\;\left(\text{score}\right)\;\text{vector},\;\text{and}$$

$$H{\left(\hat{\beta }\right)}^{\left(t\right)}=\frac{\partial^{2}L\left(\beta \right)}{\partial \beta \partial \beta^{\prime }},\;\text{is}\;\text{Hessian}\;\text{matrix}\;\text{of}\;\text{the}\;\text{log}\;\text{likelihood}\;\text{function},$$

where both are evaluated at the t-th iteration.

### Convidence interval

Suppose given random sample $\left\{(x_{1i},\ldots ,x_{pi}),\;i=1,2,..,n\right\}$ that is assumed to be independent and has probability density function, where $\left(x_{1i},\ldots ,x_{pi}\right)=x_{i}$ denotes the p-dimensional covariate vector for the i-th observation. Let $Z_{j}$​ be a standardized statistic. For a given significance level α, the corresponding the paired statistics c and d satisfy the probability equation [17]:

(9) $$P\left(c\leq Z_{j}\;\leq d\right)=1-\propto$$

Under standard regularity conditions, the maximum likelihood estimator $\hat{\beta }$ is asymptotically normally distributed [17], such that

(10) $$\sqrt{n}\left(\hat{\beta }-\beta \right)\overset{d}{\to }N\left(0,{\left[I\left(\hat{\beta }\right)\right]}^{-1}\right)\;or\;\hat{\beta }\;\approx \;N\left(\beta ,\;{\left[{\left[I\left(\hat{\beta }\right)\right]}^{-1}\right]}^{-1}\right)$$

Note that, here, the Fisher information matrix is given by

$$I\left(\hat{\beta }\right)=\;X`\mathrm{WX}\text{,}\;\text{with}\;W=\;diag\left[\pi \left(x_{i}\right)\;\left(1-\;\pi \left(x_{i}\right)\right)\right],$$

since the parameter estimator is asymptotically normally distributed and its variance is obtained from the inverse Fisher information matrix, the confidence interval is constructed using the standard normal distribution.

#### Pivotal quantity

The purpose of the pivotal quantity is to construct a confidence interval for the unknown parameter. According to Eq. (10), the following statistics can be defined

(11) $$Z_{j}∼\;N\left(0,1\right),\;\;Z_{j}=\frac{{\hat{\beta }}_{j}-\beta_{j}}{\hat{SE}\left({\hat{\beta }}_{j}\right)},\;j=1,2,\ldots ,p$$

where $\hat{SE}\left({\hat{\beta }}_{j}\right)=\sqrt{{\left(X^{\prime }WX\right)}_{jj}^{-1}}$ denotes the standard error estimator.

In this section, it should be noted that we determine the asymptotic properties of the parameter and probability estimators. Using the properties of the Newton Raphson algorithm and the Continuous Mapping Theorem (CMT), we show that the estimated parameter vector $\hat{\beta }$ and the estimated outcome probability $\hat{\pi }\left(x_{i}\right)$ are consistent estimators of the true parameter vector β and the true outcome probability $\pi (x_{i})$, respectively.

#### Theorem 1

The Eq. (11) can be used as a pivotal quantity that the following conditions hold:

C1 (Consistency of the estimators): ${\hat{\beta }}_{j}\overset{P}{\to }\beta_{j},\;$and $\hat{\pi }\left(x_{i}\right)\overset{P}{\to }\pi \left(x_{i}\right),\;n\to \infty$

C2 (Consistency of the standard error estimator): $\hat{SE}\left({\hat{\beta }}_{j}\right)\overset{P}{\to }\sigma_{j}=SE\left(\beta_{j}\right),\;n\to \infty$. where $\sigma_{j}=\sqrt{\sigma_{j}^{2}\;}$ denotes the asymptotic standard error of ${\hat{\beta }}_{j}$.

Under these two conditions given above, the following applies asymptotically [18].

If ${\hat{\beta }}_{n}\overset{P}{\to }\beta$ and g is continue function, so $g\left({\hat{\beta }}_{n}\right)\overset{P}{\to }g\left(\beta \right)$ is valid

If $X_{n}\overset{d}{\to }x$ and $Y_{n}\overset{P}{\to }a$, so $X_{n}Y_{n}\overset{d}{\to }xa$

$$c)\;\sqrt{n}\left({\hat{\beta }}_{j}-\beta_{j}\right)\overset{d}{\to }N(0,\sigma_{j}^{2},\;\text{then}\;\frac{\left({\hat{\beta }}_{j}-\beta_{j}\right)}{\hat{SE}\left({\hat{\beta }}_{j}\right)}\;\overset{d}{\to }N\left(0,1\right)$$

Consequently, Eq. (11) defines an asymptotically valid pivotal quantity for constructing confidence intervals for $\beta_{j}$.

#### Proof C1

Let ${\hat{\beta }}_{j}\;$be the estimator of $\beta_{j}$ obtained via the Newton–Raphson algorithm. The Newton–Raphson iteration is given by

$${\hat{\beta }}^{\left(t+1\right)}={\hat{\beta }}^{\left(t\right)}-{\left[H\left({\hat{\beta }}^{\left(t\right)}\right)\right]}^{-1}g\left({\hat{\beta }}^{\left(t\right)}\right).$$

Taking probability limits (plim) on both sides yields

$$\underset{n\to \infty }{plim}\;{\hat{\beta }}^{\left(t+1\right)}=\underset{n\to \infty }{plim}\left({\hat{\beta }}^{\left(t\right)}-{\left[H\left({\hat{\beta }}^{\left(t\right)}\right)\right]}^{-1}g\left({\hat{\beta }}^{\left(t\right)}\right)\right)$$

$$\underset{n\to \infty }{plim}\;{\hat{\beta }}^{\left(t+1\right)}=\underset{n\to \infty }{plim}\;{\hat{\beta }}^{\left(t\right)}-\underset{n\to \infty }{plim}\;{\left[H\left({\hat{\beta }}^{\left(t\right)}\right)\right]}^{-1}g\left({\hat{\beta }}^{\left(t\right)}\right)$$

By the CMT and standard regularity conditions,

(12) $$\underset{n\to \infty }{plim}\;{\hat{\beta }}^{\left(t+1\right)}=\beta^{\left(t\right)}-{\left[H\left({\hat{\beta }}^{\left(t\right)}\right)\right]}^{-1}g\left(\beta^{\left(t\right)}\right)=\beta^{\left(t+1\right)}$$

Therefore, we validate that the estimated parameter vector ${\hat{\beta }}^{\left(t+1\right)}$ is a consistent estimator of the true parameter vector $\beta^{\left(t+1\right)}$, such that ${\hat{\beta }}^{\left(t+1\right)}\overset{P}{\to }\beta^{\left(t+1\right)}$ as n→∞. Consequently, it follows that each component $\beta_{j}$ of the vector ${\hat{\beta }}^{\left(t+1\right)}$ is a consistent estimator of its corresponding true parameter $\beta_{j}$, i.e., ${\hat{\beta }}_{j}\overset{P}{\to }\beta_{j}\;\text{when}\;n\to \infty$.

Furthermore, since ${\hat{\beta }}_{j}$ is a consistent estimator for $\beta_{j}$, it follows that the estimator $\hat{\pi }\left(x_{i}\right)\;$converges to $\pi (x_{i})$. That is, $\hat{\pi }\left(x_{i}\right)\overset{P}{\to }\pi \left(x_{i}\right)$, whenn→∞. The estimated outcome probability $\hat{\pi }\left(x_{i}\right)\;$is defined as

$$\hat{\pi }\left(x_{i}\right)=\;\frac{\exp(X^{\prime }\hat{\beta })}{1+\;\exp(X^{\prime }\hat{\beta })}$$

where $X^{T}\hat{\beta }={\hat{\beta }}_{0}+\;\sum_{j=1}^{p}\sum_{k=1}^{m}{\hat{\beta }}_{jk}x_{ji}^{k}+\;\sum_{j=1}^{p}\sum_{u=1}^{r}{\hat{\beta }}_{j(m+u)}{\left(x_{ji}-\;K_{ju}\right)}_{+}^{m}\;$, as defined in (5). Taking probability limits, we obtain

$$\underset{n\to \infty }{plim}\hat{\pi }\left(x_{i}\right)=\underset{n\to \infty }{plim}\left(\frac{e^{X^{\prime }\hat{\beta }}}{1+e^{X^{\prime }\hat{\beta }}}\right)$$

Since the logistic function is continuous, the CMT implies

(13) $$\underset{n\to \infty }{plim}\;\hat{\pi }\left(x_{i}\right)=\underset{n\to \infty }{plim}\frac{e^{X^{\prime }\hat{\beta }}}{1+e^{X^{\prime }\hat{\beta }}}=\frac{\underset{n\to \infty }{plim}\;\left(e^{X^{\prime }\hat{\beta }}\right)}{\underset{n\to \infty }{plim}\;\left(1+e^{X^{\prime }\hat{\beta }}\right)}=\frac{e^{X^{\prime }\hat{\beta }}}{1+e^{X^{\prime }\hat{\beta }}}=\pi \left(x_{i}\right)$$

It can be concluded that the estimator $\hat{\pi }\left(x_{i}\right)$ is a consistent estimator for $\pi (x_{i})$, i.e., $\hat{\pi }\left(x_{i}\right)\overset{P}{\to }\pi \left(x_{i}\right)$ as n→∞.

#### Proof C2

The variance estimator $\hat{SE}\left({\hat{\beta }}_{j}\right)$ of $\beta_{j}$ can be obtained by the following equation

$$Var\left({\hat{\beta }}_{j}\right)={\left[{\left(X^{T}\hat{W}X\right)}^{-1}\right]}_{jj}=\sum_{i=1}^{n}x_{ij}^{2}\hat{\pi }\left(x_{i}\right)\left(1-\hat{\pi }\left(x_{i}\right)\right)$$

where $\hat{W}=diag{\left[\pi \left(x_{i}\right)(1-\pi \left(x_{i}\right)\right]}_{i=1}^{n}$, as described in (10).

Equivalently, the estimated standard error of $\beta_{j}\;$can be written as

$$\hat{SE}\left({\hat{\beta }}_{j}\right)=\sqrt{\sum_{i=1}^{n}x_{ij}^{2}\hat{\pi }\left(x_{i}\right)\left(1-\hat{\pi }\left(x_{i}\right)\right)}$$

By the C1, we have $\hat{\pi }\left(x_{i}\right)\overset{P}{\to }\pi \left(x_{i}\right),\;n\to \infty$ for each i. Furthermore, since the function g is continuous on (0,1), the CMT implies

$$\hat{\pi }\left(x_{i}\right)\left(1-\hat{\pi }\left(x_{i}\right)\right)\overset{P}{\to }\pi \left(x_{i}\right)\left(1-\pi \left(x_{i}\right)\right).$$

Applying Slutsky’s theorem and the CMT again yields

(14) $$\begin{array}{c} \underset{n\to \infty }{plim}\hat{SE}\left({\hat{\beta }}_{j}\right)=\underset{n\to \infty }{plim}\left(\sqrt{\sum_{i=1}^{n}x_{ij}^{2}\hat{\pi }\left(x_{i}\right)\left(1-\hat{\pi }\left(x_{i}\right)\right)}\right)\\ \;=\sqrt{\sum_{i=1}^{n}x_{ij}^{2}\underset{n\to \infty }{plim}\;\left[\hat{\pi }\left(x_{i}\right)\;\left(1-\hat{\pi }\left(x_{i}\right)\right)\right]}\\ =\sqrt{\sum_{i=1}^{n}x_{ij}^{2}\pi \left(x_{i}\right)\left(1-\pi \left(x_{i}\right)\right)}\;=\sigma_{j} \end{array}$$

Hence, $\hat{SE}\left({\hat{\beta }}_{j}\right)\overset{P}{\to }\sigma_{j}=SE\left(\beta_{j}\right),$ as n→∞.

This completes the proof. Moreover, the estimator $\hat{SE}\left({\hat{\beta }}_{j}\right)$ is a consistent estimator for $SE(\beta_{j})$. Therefore, from Eq. (12–14), it follows that the statistic $Z_{j}$ is asymptotically standard normal. Consequently, $Z_{j}$ can be used as a pivotal quantity for constructing confidence intervals for $\beta_{j}$.

#### Lagrange function

Using the pivotal quantity, the values of c and d that satisfy Eq. (9) can be determined.

$$P\left(c\leq \frac{{\hat{\beta }}_{j}-\beta_{j}}{\hat{SE}\left({\hat{\beta }}_{j}\right)}\;\leq d\right)=1-\alpha$$

Equivalently,

$$P\left(c\;\hat{SE}\left({\hat{\beta }}_{j}\right)\leq {\hat{\beta }}_{j}-\beta_{j}\;\leq d\;\hat{SE}\left({\hat{\beta }}_{j}\right)\right)=1-\alpha ,$$

that can be reimpressed as

$$P\left({\hat{\beta }}_{j}-d\;\hat{SE}\left({\hat{\beta }}_{j}\right)\leq \beta_{j}\leq {\hat{\beta }}_{j}-c\;\hat{SE}\left({\hat{\beta }}_{j}\right)\right)=1-\alpha$$

The length of the above confidence interval equation is defined by

$$l\left(c,\;d\right)=-c\hat{SE}\left({\hat{\beta }}_{j}\right)-\left(-d\hat{SE}\left({\hat{\beta }}_{j}\right)\right)=-c\hat{SE}\left({\hat{\beta }}_{j}\right)+\;d\hat{SE}\left({\hat{\beta }}_{j}\right)$$

$$=\left(d-c\right)\;\hat{SE}\left({\hat{\beta }}_{j}\right)$$

For example, $Z_{j}=\frac{{\hat{\beta }}_{j}-\beta_{j}}{\hat{SE}\left({\hat{\beta }}_{j}\right)}\;∼\;N\left(0,1\right)$ is pivotal quantity for $\beta_{j}$ parameter. The shortest (1−α)×100% confidence interval can be obtained by solving Lagrange function under the probability constraint Φ(d)−Φ(c)=(1−α), given by

(15) $$L\left(c,d,\lambda \right)\;=\;\left(d-c\right)\;\hat{SE}\left({\hat{\beta }}_{j}\right)\;+\;\lambda \left[\Phi \left(d\right)\;-\;\Phi \left(c\right)-\;\left(1\;-\;\alpha \right)\right]$$

where l(c,d) is interval length and Φ(.) is cumulative distribution function (CDF) of the standard normal distribution. The Lagrange function optimization is solved by taking the partial derivatives of the Lagrange function:

(16) $$\frac{\partial L\left(c,d,\lambda \right)}{\partial c}=-\hat{SE}\left({\hat{\beta }}_{j}\right)-\;\lambda \emptyset \left(c\right)=0$$

(17) $$\frac{\partial L\left(c,d,\lambda \right)}{\partial d}=\hat{SE}\left({\hat{\beta }}_{j}\right)+\;\lambda \emptyset \left(d\right)=0$$

(18) $$\frac{\partial L\left(c,d,\lambda \right)}{\partial \lambda }=\;\Phi \left(d\right)\;-\;\Phi \left(c\right)-\;\left(1\;-\;\alpha \right)=0$$

where ∅(.) denotes the standard normal density function. From equations (2.15) and (2.16), it follows that

∅(d)−∅(c),whichimpliesc=−d.

Notice that under the standard normal assumption, the values of c and d are obtained directly from the quantiles of the standard normal distribution, namely $c=-Z_{\alpha \;/\;2}$ aand $=Z_{\alpha \;/\;2}$. Therefore, the shortest confidence interval for the MTSLM is given by

(19) $$P\left({\hat{\beta }}_{j}-Z_{\alpha \;/\;2}\;\hat{SE}\left({\hat{\beta }}_{j}\right)\leq \beta_{j}\leq {\hat{\beta }}_{j}+Z_{\alpha \;/\;2}\;\hat{SE}\left({\hat{\beta }}_{j}\right)\right)=1-\alpha$$

### Method validation

#### Simulation data application

**Fig. 1:**
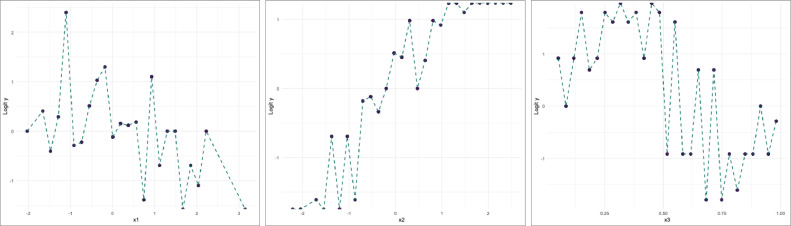
Scatterplots between HDI observed logit with each predictor variable of simulated data.

A simulation study was conducted using a nonlinear sample size of 200 to evaluate the confidence interval performance of the MTSLM, namely $x_{1},\;x_{2},\;x_{3}$. Simulated data were generated to reflect varying patterns across three predictor variables. Scatterplots were constructed by grouping the predictor values and plotting them against the logit value within each group to explore the relationship between variables. These visualizations help identify underlying patterns that are consistent with a nonparametric regression structure. The resulting scatterplots are presented in Fig. 1.

From the simulated scatterplots, there is an indication of tendency changes across different sub-intervals of the grouped predictor variables. For the $x_{1}$, a decreasing trend in the estimated effect is observed on group five, suggesting a extreme change in the relationship within that interval. For the $x_{2}$, the pattern exhibits a more pronounced variation, indicating a significant change in the response behavior across groups. Meanwhile, for the $x_{3}$, a notable shift is observed starting from group 13 and above, where the pattern becomes more distinct compared to earlier intervals. These observed suggest that the relationship between the variables is nonlinear and may differ across sub-intervals. Therefore, based on the generated data structure, a truncated spline approach is considered appropriate to capture these changes while maintaining a flexible model representation.

Given the presence of sharply changing patterns across the predictor variables, a selection of knot points is required to capture the relationship. In this study, the selection of knot points was performed by evaluating one, two, three, and four candidate knot combinations, where each combination was assessed based on Akaike Information Criterion (AIC). The optimal knot configuration is then chosen as the one that yields the minimum AIC value, indicating the best balance between model fit and complexity

The results show that the optimal number of knot points for the simulation data are $K_{1u}=$ 4, and $K_{2u}=$ 3, $K_{3u}=$ 4, with the smallest AIC value of 147.2145, as shown in Table 1. The selected optimal knot locations are obtained as follows: $x_{1}$is $K_{11}=$ 0.789, $K_{12}=$ 0.219, $K_{13}=$ 0.274, and $K_{14}=\;$0.802. For $x_{2}$ is $K_{21}=$ 0.806, $K_{22}=\;$0.423, and $K_{23}=$ 0.268. For $x_{3}$ is $K_{31}=$ 0.202, $K_{32}=$ 0.401, $K_{33}=$ 0.600, and $K_{44}=$ 0.799. Thus, the Multivariable Truncated Spline Linear Model (MTSLM) can be written as follows:

$$logit\left(\pi \left(x_{i}\right)\right)=\beta_{0}+\;\beta_{11}x_{1i}+\;\beta_{12}{\left(x_{1i}-\;0.789\right)}_{+}+\;\beta_{13}{\left(x_{1i}-0.219\right)}_{+}+\beta_{14}{\left(x_{1i}-0.274\right)}_{+}+\beta_{15}{\left(x_{1i}-\;0.802\right)}_{+}+\;\beta_{21}x_{2i}+\;\beta_{22}{\left(x_{2i}-0.806\right)}_{+}+\beta_{23}{\left(x_{2i}-0.423\right)}_{+}+\beta_{24}{\left(x_{2i}-0.268\right)}_{+}+\;\beta_{31}x_{3i}+\;\beta_{32}{\left(x_{3i}-0.202\right)}_{+}+\;\beta_{33}{\left(x_{4i}-0.401\right)}_{+}+\;\beta_{34}{\left(x_{4i}-0.600\right)}_{+}+\;\beta_{35}{\left(x_{4i}-0.799\right)}_{+}$$

**Table 1:** Determining the optimal number of knot points in the simulated data.

No.	Combination of Knot Point	AIC Value
1	1,1,1	176.0401
2	1,1,2	176.9084
3	1,1,3	167.7689
4	1,1,4	154.5854
5	1,2,1	177.9032
6	1,2,2	178.8694
7	1,2,3	168.989
8	1,2,4	155.846
9	1,3,1	178.7207
10	1,3,2	179.6977
⋮		
60	4,3,4	147.2145
61	4,4,1	176.9115
62	4,4,2	177.2112
63	4,4,3	163.5687
64	4,4,4	147.3441

The next step is to construct confidence intervals for each parameter in the model. This study uses a 95% confidence level, calculated based on Eq. (19). Conclusions are drawn if zero is not included in the interval, then the parameter is considered to have a significant effect on the model. The results of the confidence interval calculations for the MTSLM parameters are presented in Table 2 below.

**Tabel 2:** 95% Confidence interval of parameters in the MTSLM of simulated data.

Parameter	Estimate	Lower	Upper
${\hat{\beta }}_{0}$	−0.0321	3.0829	4.0186*
${\hat{\beta }}_{11}$	−1.5943	3.0396	0.8509*
${\hat{\beta }}_{12}$	−2.1378	−6.7725	2.4969
${\hat{\beta }}_{13}$	6.2965	−0.1823	12.7752
${\hat{\beta }}_{14}$	−11.3356	−19.4642	−3.2069*
${\hat{\beta }}_{15}$	10.2900	3.8685	16.7116*
${\hat{\beta }}_{21}$	2.1257	0.1073	4.1441*
${\hat{\beta }}_{22}$	−3.7589	−10.1441	2.6263
${\hat{\beta }}_{23}$	6.1714	−1.1619	13.5047
${\hat{\beta }}_{24}$	−3.7027	−7.1559	−0.2495*
${\hat{\beta }}_{31}$	15.5131	8.7983	12.2280*
${\hat{\beta }}_{32}$	−19.4373	−8.1552	−0.7195
${\hat{\beta }}_{33}$	−13.9991	−7.7507	9.7524
${\hat{\beta }}_{34}$	1.1753	−9.1454	21.4961
${\hat{\beta }}_{35}$	6.4531	1.7700	11.1361*

⁎ denotes statistically significant parameters.

From these results, it can be seen that several parameters have confidence intervals that do not include zero, so it can be concluded that these parameters are statistically significant in the model. These significant parameters are marked with an asterisk (*).

After estimating the MTSLM and interpreting its parameters, it is necessary to evaluate whether the added complexity of the model truly offers meaningful advantages. For comparison purposes, a convetional binary logistic regression model was also developed at this stage to examine the relationship between the simulation data used in this study.

$$\pi \left(x_{i}\right)=\frac{\exp(1.7697-1.4015x_{1i}-1.1023x_{2i}-2.7412x_{3i})}{1+\;\exp(1.7697-1.4015x_{1i}-1.1023x_{2i}-2.7412x_{3i})}$$

The following table lists a comparison of deviance and AIC values between the conventional binary logistic regression model and the MTSLM model Table 3.

**Tabel 3:** Comparison of deviance value and AIC for simulated data.

Model	Deviance	AIC
Binary Logistic Regression	176.6742	184.6742
MTSLM	117.2145	147.2145

Based on a comparison of deviance and AIC, MTSLM performs better than binary logistic regression for the simulated data. This is evident from the smaller deviance value of MTSLM (117.2145 vs. 176.6742) and the lower AIC (147.2145 vs. 184.6742). Therefore, MTSLM can be concluded as the more optimal model for this simulated data.

Based on the classification performance evaluation results in Table 4, MTSLM demonstrated better performance than binary logistic regression across all indicators. MTSLM achieved an AUC of 94.44%, which is higher than the logistic model’s 82.55%, indicating better discriminatory ability in distinguishing between classes. Additionally, MTSLM also yields higher accuracy (87%), sensitivity (89.38%), specificity (83.9%), and F1 score (88.59%) values compared to binary logistic regression. This indicates that MTSLM is not only more accurate overall but also more balanced in identifying both classes, positive and negative.

**Table 4:** Calculation of model classification goodness for simulated data.

Model	AUC	Accuracy	Sensitivity	Specificity	F1 Score
Binary Logistic Regression	82.55%	72.5%	77.31%	68.71%	76.09%
MTSLM	94.44%	87%	89.38%	83.9%	88.59%

#### Real data application

In applying the MTSLM method, this study uses secondary data on the status of regions based on the HDI in the year. The research variables used in this study are presented in Table 5.

**Tabel 5:** Research variables.

Notation	Variable	Desctiprion
y	Human Development Index (HDI)	0: Low HDI, 1: High HDI
$x_{1}$	Percentage of Households with Access to Clean Water	Ratio
$x_{2}$	Percentage of Poor Population	Ratio
$x_{3}$	Open Unemployment Rate	Ratio
$x_{4}$	High School Enrollment Rate	Ratio

Based on data from 514 districts/cities in Indonesia in 2023 that were sampled for the study, there were 305 districts/cities in the high HDI category (HDI ≥ 70). Furthermore, there were 206 districts/cities in the low HDI category (HDI < 70) [19]. The multicollinearity test is shown in the Table 6 below.

**Tabel 6:** VIF.

Variable	VIF
$X_{1}$	1.041347
$X_{2}$	−0.1854
$X_{3}$	0.2643
$X_{4}$	0.2636

**Fig. 2:**
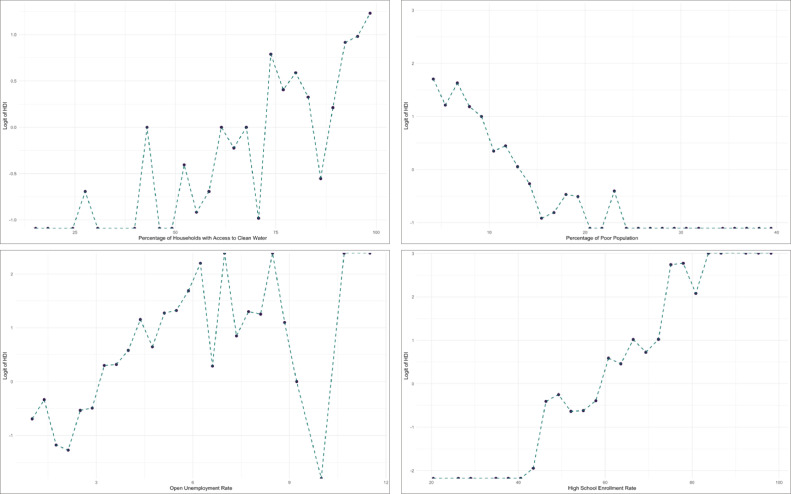
Scatterplot between HDI observed logit with each predictor variable of HDI data.

Based on Table 6, each predictor variable has a VIF value of <10, so it can be concluded that there is no multicollinearity between the predictor variables in the model. To explore the potential nonlinear relationships between each predictor and HDI status, we constructed scatterplots of grouped data against the Logit of HDI. These are presented in Figure 2.

Based on the scatter plot in Fig. 2, it can be initially identified that there is a tendency for the relationship to change sharply at certain sub-intervals between the HDI logit and the predictor variables. For the access to clean water variable, a pronounced change in the pattern is detected around the group corresponding to a value of 75. Similarly, the percentage of poor population exhibits varying tendencies across certain sub-intervals. For the unemployment rate, a more evident structural change is observed toward the upper tail of the data. Meanwhile, the school enrollment rate shows noticeable fluctuations, particularly around the intervals near values of 50 and 70, where the trend changes more distinctly compared to adjacent regions. Therefore, in this study, HDI modeling is conducted using a linear truncated spline (m=1) approach, which is suitable for capturing local changes in the relationship while maintaining model interpretability. To validate the proposed MTSLM method, the dataset was randomly divided into training and testing subsets. 80% of the data were used for model estimation (training set), while the remaining 20% were reserved for model evaluation (testing set).

The analysis then proceeds by applying the model to the complete dataset with all knot point combination, with the number of knot points on each predictor variable limited to a maximum of four. The selection of the optimal knot points using the minimum AIC value. The optimal number of knot points for HDI data, are $K_{1u}=$ 1, $K_{2u}=$ 1, $K_{3u}=$ 1, and $K_{4u}=$ 4, where the minimum AIC value is 345.637 as presented in Table 7 below:

**Table 7:** Determining the optimal number of knot points in the HDI data.

No.	Combination of Knot Point	AIC Value
1	1,1,1,1	346.6438
2	2,1,1,1	348.621
3	3,1,1,1	349.5365
4	4,1,1,1	349.7302
5	1,2,1,1	348.2156
6	2,2,1,1	350.1962
7	3,2,1,1	351.0136
⋮		
193	1,1,1,4	345.637
194	2,1,1,4	347.5948
195	3,1,1,4	348.5591
196	4,1,1,4	348.6999
197	1,2,1,4	346.8317
198	2,2,1,4	348.7873
199	3,2,1,4	349.6076
200	4,2,1,4	349.7587
⋮		
255	3,4,4,4	358.2794
256	4,4,4,4	358.2481

Based on Table 7 the optimal model obtained, it can be seen that the optimal knot point model for $X_{1}$is $K_{11}=$ 91.13, for $X_{2}$ is $K_{21}=$ 9.53, for $X_{3}$ is $K_{31}=$ 4.05, and, for $X_{4}$ are $K_{41}=$ 58.652, $K_{42}=$ 62.578, $K_{43}=$ 64.518, and $K_{44}=$ 67.634. So that the MTSLM can be expressed as follows:

$$logit\left(\pi \left(x_{i}\right)\right)=\beta_{0}+\;\beta_{11}x_{1i}+\;\beta_{12}{\left(x_{1i}-\;91.13\right)}_{+}+\;\beta_{21}x_{2i}+\;\beta_{22}{\left(x_{2i}-\;9.53\right)}_{+}+\;\beta_{31}x_{3i}+\;\beta_{32}{\left(x_{3i}-\;4.05\right)}_{+}+\;\beta_{41}x_{4i}+\;\beta_{42}{\left(x_{4i}-\;58.652\right)}_{+}+\;\beta_{43}{\left(x_{4i}-\;62.578\right)}_{+}+\;\beta_{44}{\left(x_{4i}-\;64.518\right)}_{+}+\;\beta_{45}{\left(x_{4i}-\;67.634\;\right)}_{+}$$

After obtaining the MTSLM in HDI, the next step is to compile confidence intervals for each parameter in the MTSLM. In this study, a 95% confidence interval was used, which was formed using Eq. (19). Conclusions were drawn by observing whether the confidence interval of a parameter included the value zero. If the confidence interval does not contain zero, then the parameter has a significant effect on the model. The following presents the results of the confidence interval for the parameters of the MTSLM.

**Fig. 3:**
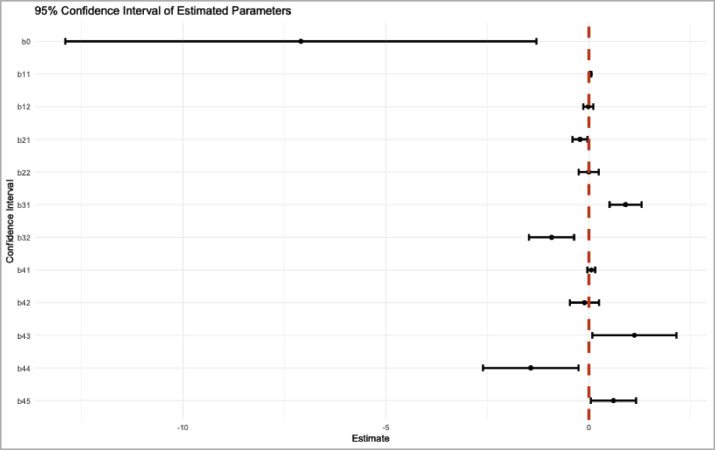
95% confidence interval plot of the estimated parameters.

Based on Table 8, it can be seen that the estimated parameters ${\hat{\beta }}_{11},\;{\hat{\beta }}_{21},\;{\hat{\beta }}_{31},\;{\hat{\beta }}_{32}\;{\hat{\beta }}_{43},\;{\hat{\beta }}_{44},\;{\hat{\beta }}_{45}$ have 95% confidence intervals that do not contain zero. For instance, ${\hat{\beta }}_{11}$ ranges from 0.0124 to 0.0566, ${\hat{\beta }}_{21}$ ranges from −0.4039 to −0.0341, ${\hat{\beta }}_{31}\;$ranges from 0.5083 to 1.2985, ${\hat{\beta }}_{32}$ ranges from −1.4760 to −0.3656, ${\hat{\beta }}_{43}$ ranges from 0.0841 to 2.1558, ${\hat{\beta }}_{44}$ ranges from −2.6122 to −0.2531, and ${\hat{\beta }}_{45}$ranges from 0.0463 to 1.1620. Therefore, it can be concluded that all predictor variables that significantly affect the HDI based on the MTSLM are the Percentage of Households with Access to Clean Water ($x_{1}$), Percentage of Poor Population ($x_{2}$), Open Unemployment Rate ($x_{3}$), and High School Enrollment Rate ($x_{4}$). This result is supported by Fig. 3, which presents a coefficient plot of the 95% confidence intervals. The visualization clearly shows that significant parameters have confidence intervals that do not cross zero, while non-significant parameters intersect the zero line. This is in line with the initial identification results through scatterplots, which show a variable relationship in certain sub-intervals between all predictor variables and the HDI logit based on the observation data.

**Tabel 8:** 95% Confidence interval of parameters in HDI data.

Parameter	Estimate	Lower	Upper
${\hat{\beta }}_{0}$	−7.0936	−12.8966	−1.2906*
${\hat{\beta }}_{11}$	0.0345	0.0124	0.0566*
${\hat{\beta }}_{12}$	−0.0151	−0.1403	0.1101
${\hat{\beta }}_{21}$	−0.2190	−0.4039	−0.0341*
${\hat{\beta }}_{22}$	−0.0035	−0.2498	0.2428
${\hat{\beta }}_{31}$	0.9034	0.5083	1.2985*
${\hat{\beta }}_{32}$	−0.9208	−1.4760	−0.3656*
${\hat{\beta }}_{41}$	0.0582	−0.0383	0.1548
${\hat{\beta }}_{42}$	−0.1101	−0.4685	0.2484
${\hat{\beta }}_{43}$	1.1200	0.0841	2.1558*
${\hat{\beta }}_{44}$	−1.4327	−2.6122	−0.2531*
${\hat{\beta }}_{45}$	0.6041	0.0463	1.1620*

⁎ denotes statistically significant parameters.

Furthermore, based on the results of parameter estimation obtained in Table 8, the interpretation of the MTSLM data can be carried out for each predictor variable by assuming that the other predictor variables are constant. The following is the interpretation:

a. Percentage of Households with Access to Clean Water (X₁)
  $$\text{logit}\left(\hat{\pi }\left(x_{i}\right)\right)\left\{ \begin{array}{c} 0.0345x_{1i},\;x_{1i}<\;91.13\\ 0.0194x_{1i}-\;1.3777,\;x_{1i}\geq \;91.13 \end{array}\right.$$

For households with access to clean water <91.13%, every 1% increase increases the relative probability of the region being in the high HDI category by exp(0.0345) = 1.0351. After exceeding 91.13%, the effect decreases, as indicated by the effective coefficient 0.0345 − 0.0151 = 0.0194, so that the relative probability increases to exp(0.0194) = 1.0196. This shows that additional increases in access to clean water at already high levels have a smaller impact on the probability of a region being in the high HDI category.

b. Percentage of Poor Population (X₂)
  $$\text{logit}\left(\hat{\pi }\left(x_{i}\right)\right)\left\{ \begin{array}{c} -0.2190x_{2i},\;x_{2i}<\;9.53\;\\ -0.2225x_{2i}-\;0.0334,\;x_{2i}\geq 9.53 \end{array}\right.$$

At a poverty rate of <9.53%, every 1% increase will reduce the relative probability of a region being in the high HDI category by exp(−0.2190) = 0.8033 times. When the percentage of poor people exceeds 9.53%, this negative effect becomes stronger with an effective coefficient of −0.2190 − 0.0035 = −0.2225, and the relative probability of a region being in the high HDI category decreases to exp(−0.2225) = 0.8005 times. This shows that an increase in the poverty rate above this threshold further reduces the probability of a region being in the high HDI category.

c. Open Unemployment Rate (X_3_)
  $$\text{logit}\left(\hat{\pi }\left(x_{i}\right)\right)\left\{ \begin{array}{c} 0.9034x_{3i},\;x_{3i}<\;4.05\;\\ -0.0174x_{3i}\;+3.7272,\;x_{3i}\geq \;4.05 \end{array}\right.$$

In regions with an open unemployment rate of <4.05%, every 1% point increase in rate increases the relative probability of the region being in the high HDI category by exp(0.9034) = 2.468 times, assuming other predictor variables remain constant. Once the exceeds 4.05%, the rate of influence changes with an effective coefficient of 0.9034 − 0.9208 = −0.0174, with a value of exp(−0.0174) = 0.9827. Although this change is statistically significant, the magnitude of the effect is practically very small, so that an increase in rate at higher levels has almost no effect on the probability of a region being in the high HDI category. This indicates a nonlinear relationship with a threshold effect between rate and the probability of a region having a high HDI.

d. High School Enrollment Rate (X4)
  $$\text{logit}\left(\hat{\pi }\left(x_{i}\right)\right)\left\{ \begin{array}{c} \begin{array}{c} 0.0582x_{4i},\;x_{3i}<\;58.652\;\\ -0.0519x_{4i}-\;6.4587,\;58.652\leq x_{4i}>62.578\\ 1.0681x_{4i}-69.9237,\;62.578\leq x_{4i}>\;64.518 \end{array}\\ -0.3646x_{4i}-104.7784,\;64.518\leq x_{4i}>\;67.634\;\\ 0.2395x_{4i}-50.3544,\;x_{4i}\geq \;67.634\; \end{array}\right.$$

In regions with an rate value of <58.652%, every 1% increase in rate tends to increase the odds of risk by exp(0.0582) = 1.0600 times, although the effect is not yet statistically significant. In the range of 58.652% to 62.578%, the effective coefficient changes to 0.0582 − 0.1101 = −0.0519, with an odds ratio of exp(−0.0519) = 0.9494, which is also insignificant. Furthermore, in the range of 62.578% - 64.518%, the effective coefficient increases to −0.0519 + 1.1200 = 1.0681, with an odds ratio of exp(1.0681) = 2.910, and the effect is statistically significant. However, in the range of 64.518% - 67.634%, there is a reversal of influence with an effective coefficient of 1.0681 − 1.4327 = −0.3646 and odds ratio exp(−0.3646) = 0.6945. For rate values above 67.634%, the effect increases again with an effective coefficient of −0.3646 + 0.6041 = 0.2395 and an odds ratio of exp(0.2395) = 1.271. These results indicate that the effect of rate on the probability of a region achieving a high HDI is nonlinear and provides an optimal impact only in certain ranges.

After obtaining the MTSLM along with the estimation results and parameter interpretation, it is important to assess whether the complexity of the model provides real benefits. As a comparison model, at this stage a conventional binary logistic regression model was constructed to model the relationship between the HDI outcomes variable and the predictor variables used in this study.

$$\pi \left(x_{i}\right)=\frac{\exp(-8.1039+\;0.0325x_{1i}-0.2123x_{2i}+0.3062x_{3i}+0.1100x_{4i})}{1+\;\exp(-8.1039+\;0.0325x_{1i}-0.2123x_{2i}+0.3062x_{3i}+0.1100x_{4i})}$$

The following table lists a comparison of deviance and AIC values between the conventional binary logistic regression model and the MTSLM model.

Based on Table 9, it can be seen that the nonparametric approach produces a better model for HDI modeling than the binary logistic. Next, performance measures derived from the confusion matrix are computed to evaluate how well each model discriminates between high and low HDI. The results are summarized in Table 6.

**Tabel 9:** Comparison of deviance value and AIC for HDI data.

Model	Deviance	AIC
Binary Logistic Regression	341.119	351.118
MTSLM	321.637	345.637

Based the classification performance results in Table 10, MTSLM demonstrates higher overall classification ability compared to conventional logistic regression, as evaluated using training and testing data. The MTSLM yielded a higher Area Under the Curve (AUC) of 89.20% than the logistic model at 88.77%, indicating a better overall discriminative capability in distinguishing regions with category across different classification thresholds. In addition, the specificity of MTSLM at 75.56% is also higher than the logistic model at 73.34%, indicating a better ability to recognize areas with low HDI. The superiority of MTSLM is also reflected in the F1 score of 84.03%, which are higher than the logistic model.

**Table 10:** Calculation of model classification goodness for HDI data.

Model	AUC	Accuracy	Sensitivity	Specificity	F1 Score
Binary Logistic Regression	88.77%	79.90%	86.20%	73.34%	83.34%
MTSLM	89.20%	81.55%	86.20%	75.56%	84.03%

## Summary

The Multivariable Truncated Spline Logistic Model (MTSLM) demonstrates superior overall performance compared to the conventional binary logistic model. This superiority is indicating that MTSLM provides better discrimination and a more balanced classification between regions with high and low HDI. The improved performance highlights the advantage of incorporating truncated spline components, which allow the model to capture changing patterns on certain sub-intervals relationships that cannot be represented by standard logistic regression.

A novel contribution of this study lies in the construction of confidence intervals for the MTSLM parameters, developed using a pivotal quantity based approach. This construction enables reliable statistical inference by explicitly accounting for the uncertainty of spline based parameter estimates. A simulation study was conducted to evaluate the performance of the proposed method under controlled conditions. The result demonstrate that MTSLM performs better than standard logistic regression, thereby supporting the effectiveness of the proposed approach. In read data application, the confidence interval analysis confirms that all four predictors $x_{1}$ (Percentage of Households with Access to Clean Water), $x_{2}$ (Percentage of Poor Population), $x_{3}$ (Open Unemployment Rate), and $x_{4}$ (High School Enrollment Rate) are statistically significant in explaining the probability of achieving high HDI status. Access to clean water and high school enrollment rate, emphasizing the importance of basic infrastructure and education in human development. In contrast, the percentage of poor population and open unemployment rate, reflecting structural socioeconomic constraints that hinder HDI improvement.

Overall, these findings indicate that MTSLM not only outperforms binary logistic regression in classification accuracy but also provides a more flexible and informative representation of the underlying social economic factors influencing HDI. Despite its contributions, future research may consider extending the MTSLM framework to spatial or panel data settings in order to better account for regional interdependencies and temporal dynamics. In addition, integrating MTSLM with artificial intelligence based prediction approaches could further improve model robustness and enhance its ability to capture complex and evolving patterns of regional inequality.

## Limitations

1. Data Splitting:

The dataset is divided into training and testing sets with an 80:20 ratio. This partition may lead to slight variations in model performance across different splits.

2. Knot Selection:

The number of knots in the spline is limited to a maximum of four, selected based on the Akaike Information Criterion (AIC). Different numbers of knots may also be considered in future studies to provide additional insights and to further explore the model’s with respect to knot specification.

3. Parameter Estimation:

Model parameters are estimated using Maximum Likelihood Estimation (MLE), followed by iterative with the Newton Raphson method.

4. Classification Threshold:

The predicted probabilities are classified into 0 or 1 using a cutoff threshold of 0.5.

5. Confidence Intervals:

Confidence intervals are constructed using the Pivotal Quantity approach.

6. Data Source:

The analysis uses secondary data on the Human Development Index (HDI) at the districs/cities in Indonesia for 2023.

7. Multicollinearity Check:

Continuous predictor variables are verified to not exhibit multicollinearity.

## Related research article

For a published article: A. S. Suriaslan, I. N. Budiantara, and V. Ratnasari, “Nonparametric regression estimation using multivariable truncated splines for binary response data,” MethodsX, vol. 14, Jun. 2025, https://doi.org/10.1016/j.mex.2024.103084.

## Ethics statements

This research uses secondary data that we collected from publications issued by the Indonesian Central Statistics Agency (BPS).

## CRediT author statement

Afiqah Saffa Suriaslan: Conceptualization, Methodology, Software, Writing – original draft, Visualization.

I Nyoman Budiantara: Conceptualization, Methodology, Writing – review & editing, Validation, Supervision.

Vita Ratnasari: Conceptualization, Methodology, Validation, Supervision.

Dursun Aydin: Methodology, Validation, Formal analysis, Writing – review & editing, Supervision.

## Declaration of competing interest

The authors declare that they have no known competing financial interests or personal relationships that could have appeared to influence the work reported in this paper.

## Data Availability

I have share the link to my data and analysis code
